# Dynamic Membranes for Enhancing Resources Recovery from Municipal Wastewater

**DOI:** 10.3390/membranes12020214

**Published:** 2022-02-12

**Authors:** Pau Sanchis-Perucho, Daniel Aguado, José Ferrer, Aurora Seco, Ángel Robles

**Affiliations:** 1 CALAGUA—Unidad Mixta UV-UPV, Departament d’Enginyeria Química, Universitat de València, 46100 Burjassot, Spain; aurora.seco@uv.es (A.S.); angel.robles@uv.es (Á.R.); 2 CALAGUA—Unidad Mixta UV-UPV, Institut Universitari d’Investigació d’Enginyeria de l’Aigua i Medi Ambient—IIAMA, Universitat Politècnica de Valencia, 46100 Burjassot, Spain; daaggar@hma.upv.es (D.A.); jferrer@hma.upv.es (J.F.)

**Keywords:** direct membrane filtration, dynamic membranes, resource recovery, municipal wastewater treatment

## Abstract

This paper studied the feasibility of using dynamic membranes (DMs) to treat municipal wastewater (MWW). Effluent from the primary settler of a full-scale wastewater treatment plant was treated using a flat 1 µm pore size open monofilament polyamide woven mesh as supporting material. Two supporting material layers were required to self-form a DM in the short-term (17 days of operation). Different strategies (increasing the filtration flux, increasing the concentration of operating solids and coagulant dosing) were used to enhance the required forming time and pollutant capture efficiency. Higher permeate flux and increased solids were shown to be ineffective while coagulant dosing showed improvements in both the required DM forming time and permeate quality. When coagulant was dosed (10 mg L^−1^) a DM forming time of 7 days and a permeate quality of total suspended solids, chemical oxygen demand, total nitrogen, total phosphorous and turbidity of 24 mg L^−1^, 58 mg L^−1^, 38.1 mg L^−1^, 1.2 mg L^−1^ and 22 NTU, respectively, was achieved. Preliminary energy and economic balances determined that energy recoveries from 0.032 to 0.121 kWh per m^3^ of treated water at a cost between €0.002 to €0.003 per m^3^ of treated water can be obtained from the particulate material recovered in the DM.

## 1. Introduction

The municipal wastewater (MWW) management paradigm has changed considerably in recent years. Due to increasing worldwide water demands, the global energy crisis and climate change, the need to find new fresh water sources and develop more energy-efficient technologies with a low environmental impact is becoming imperative to ensure sustainable global economic models. As a result, numerous studies are recommending changing the current development models for new ones based on the circular economy (CE) [1]. MWW is thus beginning to be considered a relevant source of essential resources, including reclaimed water, energy and nutrients (mainly nitrogen and phosphate) [2]. Unfortunately, the current municipal wastewater treatment plants (MWWTP) fail to recover all the potential resources in MWW, focusing on managing sewage as their only goal. In fact, classical aerobic technology, which is the core of the water line treatment, is usually identified as an inefficient system, representing up to 50% of the total energy requirements of full-scale MWWTPs [3].

Several alternatives have been proposed to transform the current MWWTPs to new resource recovery facilities (e.g., the direct treatment of MWW in anaerobic membrane bioreactors [4]). However, their implementation could represent significant economic investments due to radical changes in the MWW treatment structural scheme and operating conditions. Because of that, the filtration of the MWW before the aerobic treatment, which is commonly known as direct membrane filtration (DMF), have recently been proposed as an interesting option [5]. DMF consists of using a membrane filtration system to treat raw influent MWW (after conventional screening, sieving, desanding and degreasing pre-treatment) and capture the particulate fraction of the influent sewage. Thanks to this, the oxygen demands of the biological process can be significantly reduced by lowering the organic loading rate, which in turn reduces the energy demands. Indeed, aerobic treatment could even be unnecessary depending on the generated permeate quality [6]. On the other hand, the organic material recovered in the membrane tank can be transformed into methane via anaerobic digestion (AD), enhancing the overall energy balance of the facility. The nutrient content in the particulate fraction of the MWW is also recovered in the concentrated sludge, thus improving the overall resource recovery potential during the MWW treatment without requiring a great deal of structural modifications to the current installations.

Many authors have studied different membrane technologies (i.e., microfiltration (MF), ultrafiltration (UF), nanofiltration (NF) and forward osmosis (FO) membranes) for the DMF of MWW, usually finding severe membrane fouling during the filtration process [7,8]. In fact, the development of effective and energy-efficient fouling control strategies is one of the major issues to be addressed to enhance DMF feasibility [9]. To overcome this issue, some authors have proposed using dynamic membranes (DM), showing promising results [10,11,12]. DMs consist of the formation of a cake layer on a low filtration-resistance supporting material, making the cake layer formed the main filtering actor [13]. Filtration resistance during DM operation can thus be easily controlled by physical cleaning methods and usually achieve significantly lower filtration resistance than those of other membrane technologies [10]. Additionally, the required supporting structures are generally low-cost materials such as filter-cloths and woven meshes, representing low investment cost for their acquisition and/or replacement [14]. Two different kinds of DMs can be defined depending on how the filtering cake layer is formed—namely, self-forming and pre-coated DMs [13,15]. Self-forming DMs are formed by developing the cake layer from the direct deposition onto the supporting structure of the suspended material and high molecular weight organics contained in the treated liquor while performing the filtration process. On the other hand, pre-coated DMs are formed by passing an external solution containing one or more particulate materials through the supporting structure, such as powdered carbon or kaolite, for pre-forming a stable structure onto which the filtering cake layer will be formed. In comparison, self-forming DMs are more advantageous since they do not require additional chemical dosing, reducing the operating cost [15]. However, pre-coated DMs are essential in some scenarios for reducing the DM forming time and for allowing its formation when not enough particulate material is transported by the treated liquor.

Different strategies have been proposed to improve filtration performance of DM systems when treating MWWs. Among them, coagulant dosing is one of the most recommended for reducing membrane fouling and enhancing permeate quality [8,16], helping also in the formation of the DM as a pre-coating material. Several studies agree in recommending inorganic polyaluminum chloride (PACl) coagulants [16,17], which can efficiently flocculate the small-size-range particles present in the MWW, and even capture a significant percentage of the colloidal fraction. Phosphate can also be efficiently captured via chemical precipitation, while stronger and more resistant flocs have been reported to be formed when using PACl, achieving better performances than other coagulants when carrying out filtration processes [18,19]. Another possible alternative to enhance DM resource capture efficiency is to raise the operating solids concentration, which could promote the formation of thicker and less porous cake layers on the supporting material. This would represent an interesting approach since additional operating chemicals and environmental costs could be avoided. However, increasing operating solids concentration could also raise the sludge filtering resistance, which could dramatically affect the filtration energy demand, achieving counterproductive effects. Using pre-treated influents has also been suggested to reduce the severe fouling reported when treating raw MWWs [20]. In this regard, primary settler effluent (PSE) as the DM influent could be an interesting option since a large fraction of the influent particulate material would be recovered and concentrated in a pre-treatment step, presumably reducing the fouling potential of the treated MWW. Additionally, the recovered sludge would be used in the AD in much the same way as conventional MWW treatment schemes, thereby not affecting the energy balance. However, removing the higher size particles when using DMs could also involve important negative effects on the DM self-forming capacity, DM structure and toughness, and permeate quality, which could compromise their applicability.

The aim of this work was therefore to assess the potential benefits of DMs in treating PSE from a MWWTP with a preliminary (pilot scale) study to determine the best operating conditions to carry out the filtration process. The effect of using an additional supporting material layer, increasing the filtration flux (15 and 45 LMH), different operating total suspended solids (TSS) concentrations and coagulant dose were evaluated. All the experiments focused on determining: (1) DM forming capacity when treating this influent, (2) the resource recovery capacity of the system and (3) the fouling rate and preliminary economic costs of the process.

## 2. Materials and Methods

### 2.1. Influent and Experimental Design

The influent MWW used was PSE from the full-scale “Conca del Carraixet” WWTP (Alboraya, Spain) (see the main characteristics of this influent MWW in Table 1). Two different systems (a pilot-scale plant and a lab-scale membrane module) were used to evaluate the effect of different operating conditions on the DM performance. The pilot plant was used to assess the effect of the number of supporting layers, operating flux and coagulant dosing and the lab-scale module to evaluate the effect of the operating TSS concentration (performed at lab-scale due to the difficulty of reaching the required TSS concentrations in the pilot plant). Table 2 shows the experimental conditions of every experimental period. A flat polyamide open monofilament woven mesh of 1 µm average pore size (NITEX^®^, SEFAR) was used as supporting material in all the experiments and the membrane surface was cleaned by brushing it with tap water as required.

### 2.2. DM Pilot Plant

**Figure 1:**
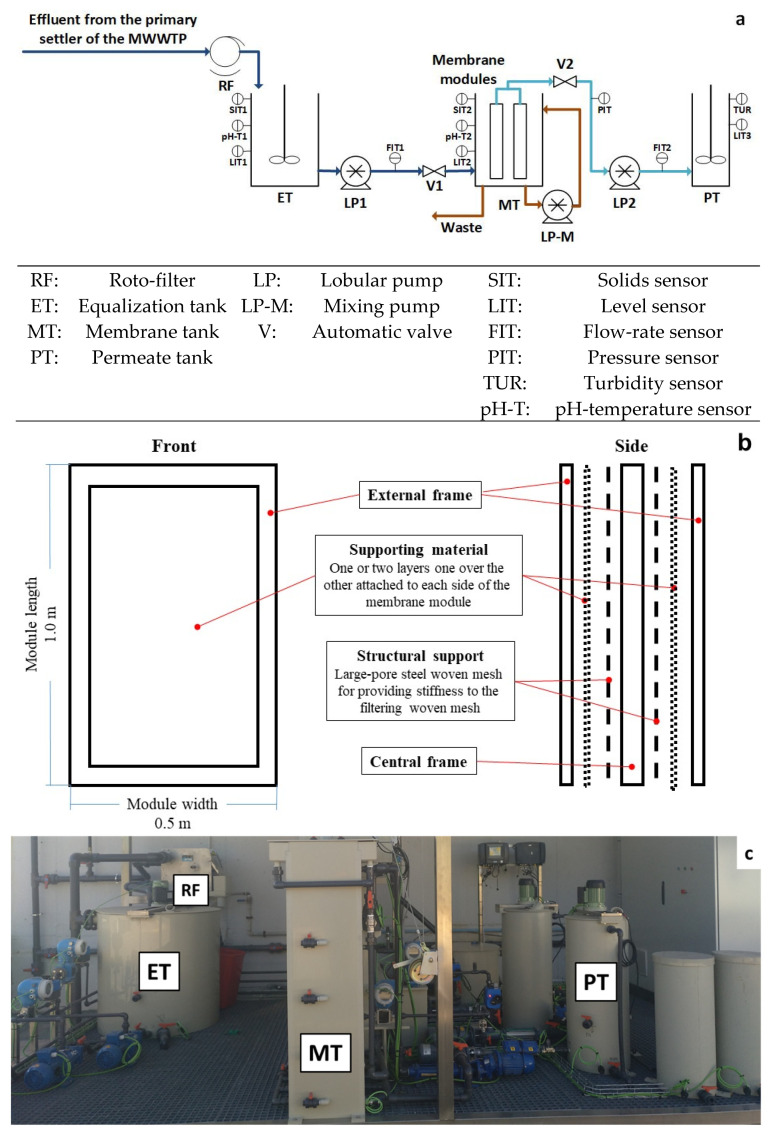
DM pilot-plant: (**a**) diagram scheme, (**b**) membrane module scheme and (**c**) picture.

Figure 1a shows a flow diagram of the DM pilot plant, which mainly consisted of a membrane tank (MT) (190-L working volume) equipped with two submerged flat membrane modules. To allow for the proper development of the DM on the supporting material, the employed woven mesh was attached to a rectangular supporting frame (1-m high and 0.5-m wide) to stiffen the supporting material. A large-pore steel woven mesh was added under each textile layer to stiffen the supporting material during filtration. The supporting frames were designed with two external surfaces open to the treatment liquor. One woven mesh was attached to each frame face, recovering the generated permeate in the interstitial space. This design allowed us to increase the membrane areas, providing a total filtration area of 2 m^2^ (Figure 1b shows a membrane module schematic draw).

The pilot plant was operated continuously at a given operating flux (see Table 2), performing filtration-relaxation cycles with a ratio of 3:1 min. The two membrane modules were connected to a lobular pump (PCM, M series, EcoMoineau™, Milano, Italy) for vacuum filtration. A lobular pump continuously mixed the concentrated sludge to ensure homogeneity in the membrane tank. The influent MWW was pre-treated with a 0.5-mm screen size roto-filter (PAM 270/500, Procesos Auto-Mecanizados, Alicante, Spain) and homogenized in a stirred equalization tank (ET) (745 L). The wasting flow was set to 2.6 L h^−1^ to prevent significant aerobic microorganism development during filtration, operating at a solids retention time (SRT) of about 3 days. When coagulant dosing was required, a peristaltic lab pump continuously injected the coagulant solution into the MT. Figure 1c shows a view of the pilot plant.

The pilot plant was equipped with several on-line sensors and automatic equipment to control and monitor all the involved variables (see Figure 1a). The on-line sensors installed were: two pH-temperature sensors (InPro3100/120/PT100, Endress+Hauser, Barcelona, Spain) in the ET and MT; three level sensors (Cerabar PMP11, Endress+Hauser, Barcelona, Spain) in each tank (ET, MT and PT); one pressure liquid sensor (IP65, Druck, TX, USA) to monitor the transmembrane pressure (TMP); two solid concentration sensors (LXV424.99.00100, Hach, Düsseldorf, Germany) in the ET and MT; and one sensor to monitor the turbidity level (LXV424.99.00100, Hach, Düsseldorf, Germany) in the PT. For actuators, the pilot plant was equipped with different frequency converters (pumps and blowers) (SINAMICS G120C, Siemens) and control valves. Plant automation was carried out by a programmable logic controller (PLC) which performed the control and data acquisition of all the instrumentation installed in the pilot plant. A SCADA system was also used to gather all the information collected and allow their proper supervision, interaction and control.

### 2.3. Lab-Scale DM

Like the pilot plant, the lab-scale DM mainly consisted of a membrane tank (8-L working volume) with submerged DM module for filtration. To allow for the proper development of the DM on the supporting material, the woven mesh was attached to a rectangular supporting frame (0.18-m height and 0.11-m width; 0.02-m^2^ total area) to provide stiffness to the woven mesh. In this case, the supporting frame was designed with only one open surface in contact with the liquor, while the other side was closed to capture the generated permeate. The lab-scale DM was operated continuously, performing the filtration process according to filtration-relaxation cycles with a ratio of 3:1 min at an operating flux of 15 LMH. The permeate was obtained by vacuum filtration using a peristaltic pump and the TMP was recorded by a pressure captor (IP65, Druck, TX, USA) installed in the permeate side. Both TMP recording and peristaltic pump control were performed by a custom-made data acquisition software, processing the input and output signals through a multichannel data acquisition card (PicoLog 1000 series, Cambridgeshire, UK). To avoid the concentration of the MWW in the membrane tank during filtration, the permeate was recycled back to the membrane tank during continuous operation, only extracting the volume required for the sampling analysis. The content of the membrane tank was completely replaced every three days with new MWW to avoid the development of any kind of microorganism and was continuously homogenized by a supplementary peristaltic pump which continuously mixed the membrane tank sludge to avoid stratification or particle sedimentation. A PVDF hollow-fiber UF membrane (0.03 µm pore size, PURON^®^ KMS, Madrid, Spain) pre-concentrated the influent MWW to feed the lab-scale unit with the TSS concentration required in each experiment (See Table 2).

### 2.4. Analytical Methods and Calculations

Influent, membrane concentrated sludge and generated permeate were sampled twice a week to evaluate the DM resource recovery capacity. The soluble fraction of the collected samples was obtained by 0.45-mm pore size membrane filtration with glass fibre filters (Millipore, Merck). Solids, total and soluble chemical oxygen demand (COD and SCOD), total and soluble nitrogen (TN and SN) and total and soluble phosphorus (TP and SP) were determined according to standard methods [21]. A laser granularity distribution analyser (Malvern Mastersizer 2000; detector range of 0.01 to 1000 µm) was employed to determine the particle size distribution of the evaluated samples. Four different PACl coagulants were tested (Feralco Iberia S.A., Alegia, Spain) (see Table 3) to determine the most suitable and its optimum dosing concentration by means of a conventional jar-test (performed according to ASTM D2035-19 standard practice).

The membrane performance analysis was studied by means of the recorded TMP, calculating the average TMP in every filtration cycle (TMP_average_), while the 20 °C-standardized operating flux (J_20_) was calculated according to the following expression:

(1) $$J_{20}=J_{T}\cdot e^{-0.0239\;\left(T-20\right)}$$

where T is the temperature and J_T_ is the imposed operating flux. The potential energy recovery from the captured COD (E_Recovered_) was calculated as follows:

(2) $$E_{\mathrm{Recovered}}\;\left({\mathrm{kWh}\;\mathrm{per}\;m}^{3}\right)={\mathrm{COD}}_{\mathrm{Influent}}\cdot {\%\mathrm{COD}}_{\mathrm{Captured}}\cdot Y^{\mathrm{CH}4}\cdot {\mathrm{CV}}_{\mathrm{CH}4}\cdot \eta_{\mathrm{CHP}}$$

where COD_Influent_ represents the COD concentration feed to the DM module (kg m^−3^), %COD_Captured_ is the percentage of COD captured by the DM during the filtration process (%), Y^CH4^ is the theoretical anaerobic methane yield of MWW sludge (3.5 × 10^−4^ m^3^ of methane per kg of COD), CV_CH4_ is the calorific power of the methane (9.13 kWh per m^3^ of methane), and η_CHP_ is the methane electricity generation efficiency of the employed CHP system. A η_CHP_ of 35% was used in this study considering the different CHP technologies currently available [22]. The energy costs were estimated at €0.07 per kWh according to current Spanish high voltage electricity rates [23,24] while the coagulant costs were estimated at €200 per ton of coagulant, according to the data provided by the supplier (Feralco Iberia S.A.).

## 3. Results and Discussions

### 3.1. Pilot Plant Operation: Effect of Operating Conditions

**Figure 2:**
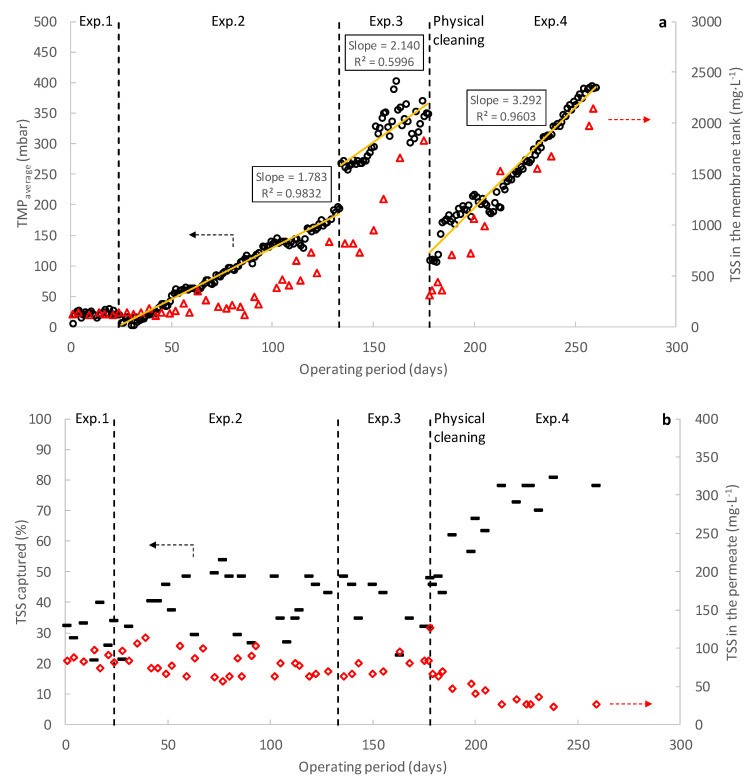
Pilot-plant performance. Evolution of (**a**) transmembrane pressure (TMP) and total suspended solids (TSS) concentration, and (**b**) TSS capture efficiency and permeate TSS concentration. 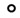 TMP_average_; 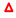 TSS in the membrane tank; 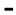 TSS capure efficiency; 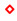 TSS in the permeate. The continuous lines represent linear fits.

**Figure 3:**
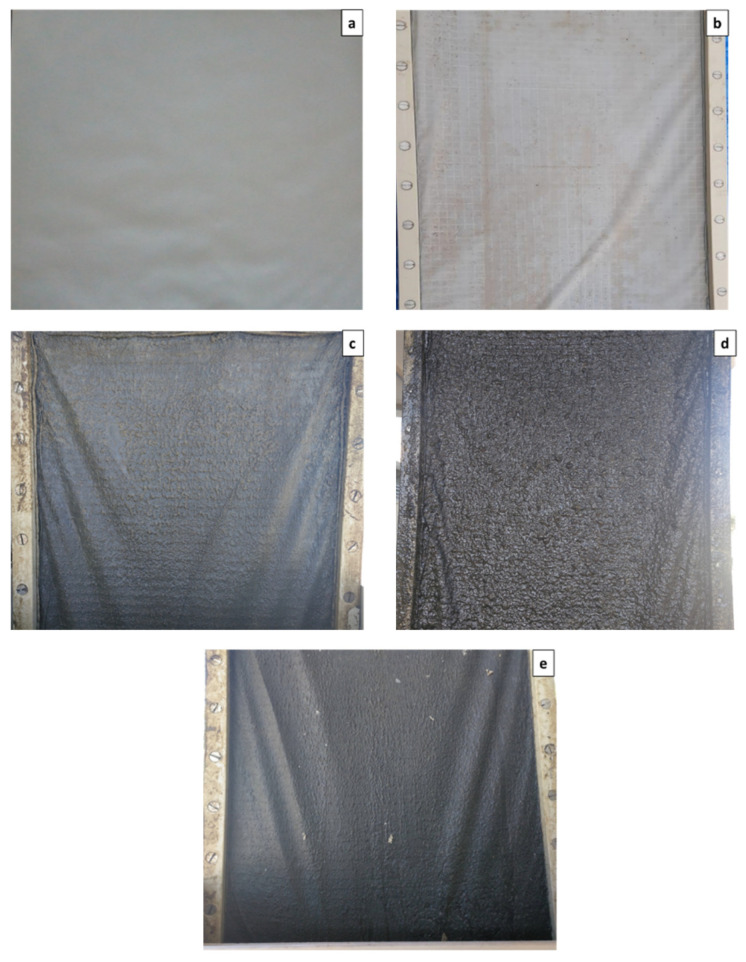
Pilot-plant supporting material after every experimental period: (**a**) New, (**b**) Exp. 1, (**c**) Exp. 2, (**d**) Exp. 3 and (**e**) Exp. 4.

Figure 2 shows the results obtained during the operation of the DM pilot plant. Exp. 1 focused on determining the possibility of self-forming a DM on the supporting material (1 µm pore size flat open monofilament woven polyamide mesh) when using effluent from the primary settler of a full-scale MWWTP. This experiment lasted for 24 days (from day 0 to 24 in Figure 2) and no significant TSS captures were detected during continuous filtration, achieving average values of about 30% (see Figure 2b). These low TSS captures were attributed to the filtering capacity of the supporting material itself, which would be able to retain mainly all the particles above 1 µm in size. Neither were any important TMPs detected during filtration, achieving values of about 20 mbar during the filtration stages (see Figure 2a), suggesting that a negligible cake layer formed on the supporting material. Since an evolution of the DM was not appreciated during the first experimental period, the membrane frame was taken out of the membrane tank to check DM development. As Figure 3b shows, very poor particle deposition was found on the supporting material after Exp. 1, showing that DM had not even started to form. Based on these results, it was concluded that the self-formation of a stable DM did not seem feasible for the supporting material and MWW studied, at least in the short-term. The results obtained during this experiment contrasted with the results reported by other studies treating MWWs by DMs, in which between 2 and 20-h self-forming times were reported using similar or even larger pore size supporting materials (between 1 and 100 µm) [10,11]. In these studies, however, raw MWW was used as influent to feed the membrane tanks, which would contain a higher amount of particulate material with a higher average particle size. Additionally, more suspended material (diatomite) was added in one of the cited studies for enhancing the DM formation [11]. All this additional particulate material would favour the development of the DM on the supporting material, especially the larger particles, which would boost the formation of a cake layer on the supporting material in the first steps. Therefore, as anticipated, the use of a more pre-treated influent, such as the one used in this study (PSE), could represent a limitation of DM applicability.

Considering the results obtained in Exp. 1, Exp. 2 was designed to enhance particle deposition on the supporting material to boost DM formation. For this, an additional woven mesh was added to each membrane frame surface, doubling their thickness. Since the new meshes were not aligned with the old, this strategy could improve DM self-forming capacity by both: (1) apparently reducing the average pore size of the supporting material, and (2) increasing the probability of contact between the threads of the woven mesh and the medium suspended particles. In fact, this strategy has been used by other authors to improve solids capture capacity [10], reducing turbidity in the generated permeate and increasing filtration resistance. Exp. 2 lasted for 108 days (from day 25 to 133), observing similar TSS captures and TMP values as those achieved in Exp. 1 during the first few days. However, after 17 days of operation (day 42), both TSS capture and TMP gradually increased daily, suggesting the development of a DM on the supporting material. Indeed, TMP steadily increased during the following 91 days (from around 20 to 190 mbar from the day 42 to 133), which could be related to the accumulation of more particles on the supporting material and the consolidation of the pre-formed DM. The TSS in the membrane tank also started a steady increase due to the enhanced DM solids capture efficiency (from around 110 to 840 mg L^−1^), which contributed to increasing TMP. However, despite the DM consolidation and the consequent rise of solid concentration in the membrane tank, TSS capture efficiency only rose to values of around 45%, reaching a pseudo-steady state after the first 18 days of operation in Exp. 2 (day 43 in Figure 2). From then on, TSS capture efficiency remained static for the rest of the period, regardless of the increase in the operating TMP or the TSS concentration in the membrane tank. Due to this low TSS capture efficiency, a relatively poor permeate quality was obtained in Exp. 2, achieving TSS, COD, TN and TP concentrations and turbidity values on the generated permeate of about 65 mg L^−1^, 141 mg L^−1^, 42.3 mg L^−1^, 4.3 mg L^−1^ and 86 NTU, respectively. Table 4 shows the permeate quality achieved during the experimental periods.

**Figure 4:**
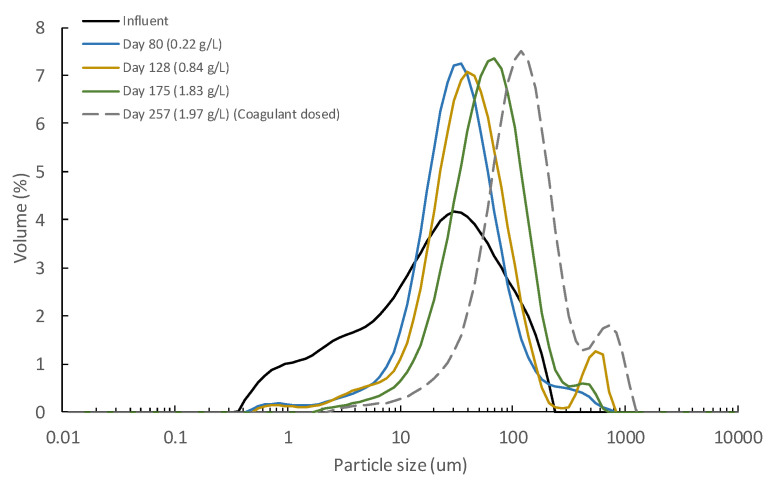
Particle size distribution of the concentrated sludge during the pilot-plant operation. Note that legend shows the day, together with the total suspended solids concentration in the pilot-plant membrane tank during sampling.

Fewer resources were recovered from Exp. 2 than in other studies treating raw MWW. Indeed, when using similar or even higher pore-sized supporting materials (between 1 and 100 µm), COD and turbidity recoveries between 63–71% and 60%, respectively, are reported in the literature [10,11]. Like the DM self-forming capacity, these different results are related to the more treated influent used in this study (i.e., PSE), which could affect resource recovery by (1) the development of a less thick DM, and (2) an intrinsic reduction of the resources that can be captured in the DM due to inclusion of the primary settler in the treatment scheme. Initially, the formation of a poor DM when using the PSE was considered, since the lower influent solids concentration could contribute to a weaker and less consolidated DM due to the reduced particle content attached to the supporting material. However, as Figure 3c shows, the DM developed during Exp. 2, although not too thick, seemed homogenous and robust enough to allow proper filtering treatment (day 133 in Figure 2). In addition, the particle size distribution analysis (see Figure 4) showed that together with the increasing TSS concentration, the average particle size of the retained particles consistently increased, which shows the DM’s capture capacity. This, together with the increased TMP during the experiment confirms that the DM formed was well developed. It was therefore assumed that the consistency of the DM during Exp. 2 was not directly related to the low resource recovery efficiencies achieved. On the other hand, despite the low resource capture capacity detected, relatively similar permeate qualities to those reported by the cited studies (i.e., [10,11]) were also obtained. The poor recovery capacity of our study could thus be due to the low suspended material loading influent treated, having captured a considerable fraction of the particulate material from the raw influent MWW in the primary settler. If this was the case, the influent used would not affect the permeate quality and the most suitable treatment scheme for full-scale implementation (i.e., direct filtration of raw MWW or the use of a primary settler as pre-treatment step) would be determined by the energy required during the filtration process in each scenario.

Considering the results obtained from Exp. 2, Exp. 3 was designed to increase the DM capture capacity. During this experimental period, the operating flux was increased from 15 to 45 LMH to favour particle deposition on the DM and induce cake compression caused by deformation of soft flocs and the structural rearrangement of particles. In fact, other authors have suggested that a more compressive and dense cake layer can be formed when filtering more treated influents due to the large number of small particles present [20,25]. Permeate quality could thus be improved by creating a dense DM with a smaller apparent pore size. Due to the significant time required to develop a consistent DM on the supporting material, Exp. 3 used the DM formed during the former experience. Exp. 3 lasted for 44 days (from day 134 to 178 in Figure 2), showing few improvements of the DM resource capture capacity (see Figure 2b and Table 4). Although the TMP rose abruptly in the first days of operation due to the larger operating flux, it then behaved like Exp. 2, which indicates similar DM filtering resistance (see Figure 2a). In fact, relatively similar fouling growth rates were achieved during the two experimental periods, obtaining a daily TMP increment of about 1.8 and 2.1 during Exp. 2 and Exp. 3, respectively. The particle size distribution (See Figure 4) and the DM physical observation after this operating period (See Figure 3d) also behaved as in Exp. 2. The results indicate that the increase in the operating flux did not alter DM morphology and constitution in the short-term. Other strategies must therefore be proposed to enhance DM capture capacity.

### 3.2. Pilot Plant Operation: Coagulant Dosing

**Figure 5:**
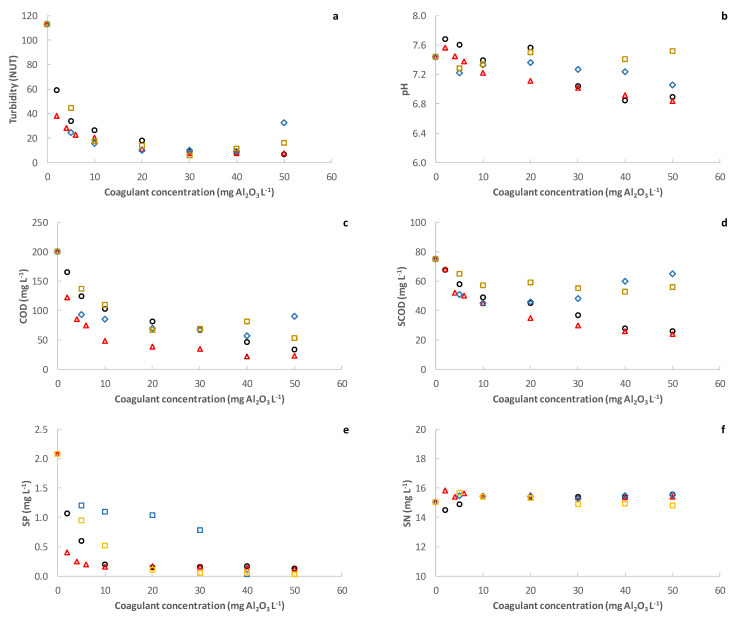
Effect of coagulant dosing on pollutant capture during jar-test: (**a**) turbidity, (**b**) pH, (**c**) chemical oxygen demand (COD), (**c**) soluble chemical oxygen demand (SCOD), (**e**) soluble phosphorus (SP) and (**f**) soluble nitrogen (SN). Coagulants used: 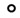 PHLA10; 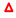 PHLA18; 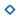 F1; 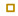 F2.

Coagulant dosing was tested as a second alternative to enhance the pilot plant’s resource recovery. Four different PACl coagulants were tested (See Table 3). The most suitable and its optimum dosing concentration was first evaluated by a conventional jar-test. As Figure 5 shows, all the employed coagulants achieved considerable pollutant captures at relatively low concentrations, including, as expected, not only a large fraction of the particulate material, but also a significant fraction of the colloidal material (which can be seen by the reduction of SCOD) and the SP. In fact, turbidity, COD, SCOD and SP reductions of up to 86, 78, 42 and 93%, respectively, were achieved during the jar-test at coagulant concentrations between 5–20 mg L^−1^. However, coagulant concentrations over 40 mg L^−1^ seemed have negative effects on solids capture in some cases, which was attributed to a destabilization of the medium charges when increasing the coagulant concentration [26]. The optimum concentration range obtained in this study was similar to that reported by other authors filtering MWW, who usually recommend PACl concentrations of around 15–30 mg L^−1^ [8,27]. On the other hand, significant SN captures were not expected or observed during this experience, since there were no relevant chemical interactions between soluble nitrogenous compounds (mainly NH_4_^+^) and the inorganic coagulants. Finally, no great pH changes were found for the coagulant concentrations tested, although a slight reduction as coagulant concentration was increased can be seen (see Figure 5). Due to the relatively high alkalinity of the MWW studied (see Table 1), this perturbation was considered negligible but could be a relevant issue in other situations. The type and optimum concentration of coagulant determined in this study could thus change in different circumstances. In this study, coagulant 2 (PHLA 18) with a concentration of 10 mg L^−1^ was chosen to operate the DM due to its slightly higher COD and SP captures than the rest of the coagulants tested.

A further experiment (Exp. 4) was then carried out focusing on the beneficial effects of continuous coagulant dosing on the DM’s performance. To properly determine the improvement in its forming time when dosing coagulant, the supporting material was physically cleaned before the experience. Exp. 4 lasted for 81 days (from day 179 to 260 in Figure 2) and a shorter forming time (of about 7 days) than Exp.2 was obtained. The operating TSS concentration and TMP increased faster during this experiment, especially in the early days. These phenomena were due to the enhanced solids capture efficiency when coagulant was dosed in the membrane tank, capturing more of the smaller particles by forming larger aggregates. Indeed, the results of the particle size distribution analysis showed a significant increase of larger particles than the influent MWW (see Figure 4). A higher amount of particulate material thus ended on the supporting material, boosting DM development. The increased solids capture capacity also accelerated the TSS concentration rate and raised the operating TMP. In this regard, other studies treating MWWs by membrane systems have showed the importance of optimizing coagulant dosing during filtration, achieving severe increases in the operating TMP with high coagulant concentrations due to the sudden accumulation of captured solids on the membrane surface [17]. As Figure 2b and Table 4 show, a significant improvement of solids capture efficiency was achieved when the coagulant was dosed. As previously mentioned, this increase was due to the capture of the small size particles, reducing significatively the turbidity of the medium. Nevertheless, as the jar-test showed (see Figure 5), coagulant dosed was unable to capture a sensible fraction of the influent colloidal material. Thus, the remaining solids detected in the permeate would be due to this colloidal fraction, together with some formed aggregates smaller than the DM average pore size, being all this particulate material able to cross through the DM and escape with the permeate. Considerable COD and TP recoveries were also achieved during Exp. 4 thanks to the capture of a fraction of the colloidal material and the chemical precipitation of phosphate when dosing the coagulant. Coagulants could thus be used to enhance MWW treatment when using DMs; however, the coagulant dosing protocol plays a critical role in the filtration process and should be carefully chosen to boost resource recovery while minimizing filtration energy demand during long-term operations. Moreover, aluminum-based coagulants, such as PACl, are usually identified as anaerobic digestion inhibitors [28], thereby reducing the energy potential of the recovered sludge. Thus, coagulant dosing minimization during filtration should be an imperative matter not only for minimizing chemicals costs, but also for avoiding recovered sludge biodegradability issues. In this regard, Hafuka et al. [29] studied the effect of PACl coagulants on the biodegradability of sludge recovered from DMF processes, reporting that Al concentrations of about 4.3 mg L^−1^ do not represent problems on the anaerobic digestion methane production. In the performed study, assuming that all the Al was captured by the membrane rejection and considering the operating permeate/waste ratio (30:2.6), Al concentrations of about 10.4 mgL^−1^ could be expected in the recovered sludge, which are not significantly superior to those reported in the cited work. Thus, no important energy recovery issues could be assumed for the recovered sludge in this case.

Finally, after Exp. 4 was concluded, the coagulant dosing was stopped, and the membrane was operated for 5 additional days to study permeate quality (data not shown). Unfortunately, a pretty similar permeate quality to those obtained in Exp. 2 and Exp. 3 was quickly achieved, showing that all the capture improvements in Exp. 4 were only due to coagulant effects and not to a change of DM structure. The visual analysis of the DM formed at the end of Exp. 4 also seemed to indicate that the DM structure remained unaltered, whatever the coagulant dosing (see Figure 3e). Nevertheless, this performance could change in long-term operations, forming a thicker and more compact DM which could itself raise the resource capture efficiency. In fact, the DM maturation period can last for several days, enhancing pollutant capture efficiency on reaching their mature state [15]. Further studies focused on dynamically optimizing the coagulant dosing protocol, considering all the important aspects (i.e., chemicals cost, filtration energy demand, resource recovery efficiency, sludge biodegradability and permeate quality) therefore need to be performed.

### 3.3. Lab-Scale Results: Effect of Solids Concentration

**Figure 6:**
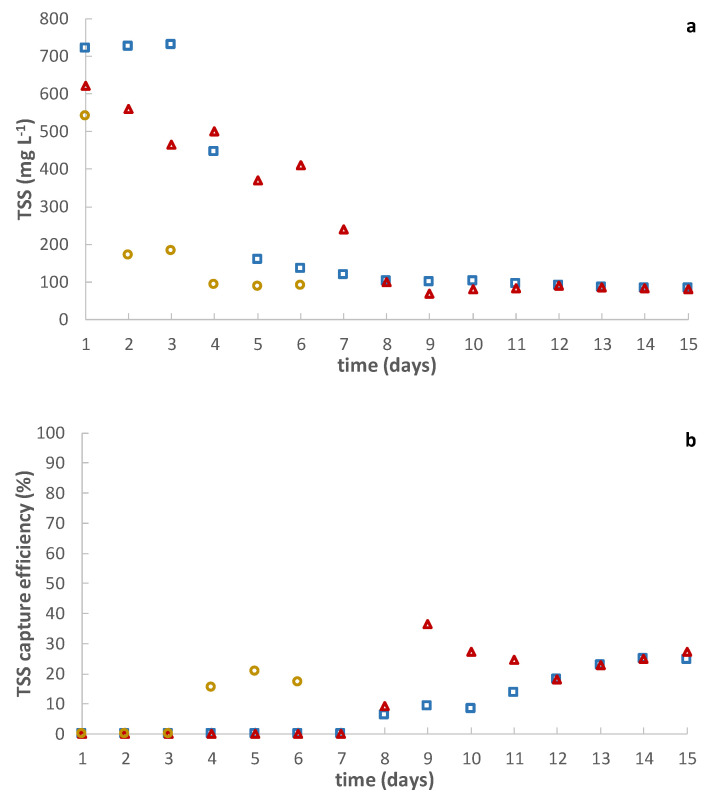
Lab-scale DM performance. Effect of influent total suspended solids (TSS) concentration (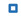 1.9 g L^−1^; 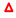 4.7 g L^−1^; 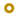 9.2 g L^−1^) on: (**a**) TSS in the permeate and (**b**) TSS capture efficiency. Note that the TSS capture efficiency was calculated based on the original influent used (see Table 1).

**Figure 7:**
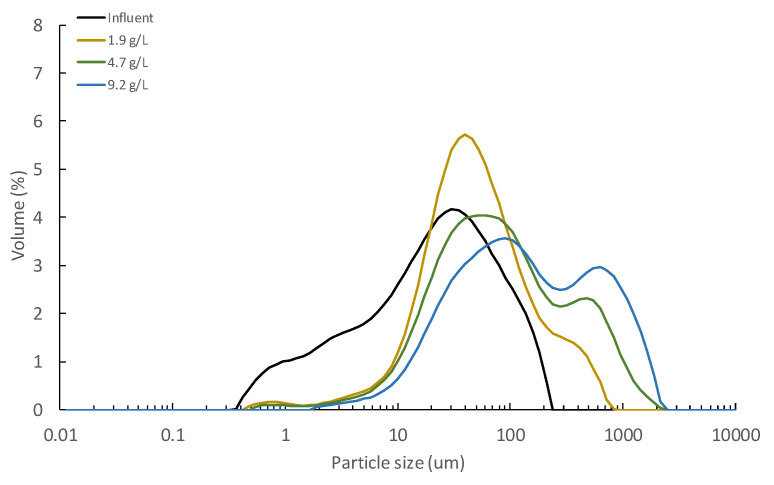
Particle size distribution of the concentrated sludge during the lab-scale operation. Note that legend shows the total suspended solids concentration fed to the lab-scale membrane which was obtained by concentrating the influent with an ultrafiltration membrane.

To discover the effect of operating TSS on short-term DM formation, the MWW used during this study was pre-concentrated to different TSS concentrations (see Table 2), and then fed to the lab-scale membrane tank before each essay. Each experiment lasted for about 15 days except for the concentration of 9.2 g L^−1^, when the experiment was stopped on the 6th day due to the severe rise of TMP. Figure 6 shows the results obtained during the lab-scale operation. The DM self-forming time onto the supporting material was significantly reduced by pre-concentrating the treated influent. In fact, self-forming times of between 4–8 days were achieved in this case, although only one supporting material layer was used. This phenomenon was associated with the higher number of particles that can be attached to the supporting material. A significant increase of the particle size distribution to higher particles sizes was also detected when concentrating the influent MWW (see Figure 7). This could be due to the sporadic flocculation of the smaller particles when increasing contact and collisions among particles at higher TSS concentrations. Higher TMPs were also obtained as the TSS concentration was raised in the membrane tank (see Table 5), which would be related with a higher accumulation of particulate material onto the formed cake layer during filtration. These results thus confirm that increasing TSS concentration is a feasible alternative to boosting DM development, but at the cost of considerably higher TMP. However, as Figure 6a shows, the permeate quality obtained regarding TSS in all the experiments was pretty similar, practically coinciding with those obtained during the pilot plant operation. Additionally, when calculating the TSS capture efficiency based on the original influent used in this study (see Table 1), low values were also obtained, regardless of the TSS concentration in the membrane tank (see Figure 6b). Therefore, these results may indicate that the solid capture efficiency of the short-term formed DM could be related higher with the influent characteristics than with the operating TSS, expecting thereby similar permeate qualities at least concerning solids’ concentration. Nevertheless, as commented above, this could significantly change in long-term operations, and further studies are required to determine the most suitable DM solids concentration when treating MWW.

### 3.4. Operating Recommendations

Aiming to roughly discern the most suitable operating approach when operating DMs with PSE as feed, a simplified economic balance was performed on every experimental period evaluated in this study, considering only energy recovery and coagulant costs. As can be seen in Table 6, there were negligible differences between Exp. 2 and Exp. 3, since the resource capture efficiency was similar in both cases. Since a similar DM formation and fouling was also found in these experimental periods (see Figure 2), increasing the operating flux as much as possible could be recommended to minimize investment and space costs as long as it does not compromise the energy required for filtration or supporting material replace periodicity. On the other hand, the enhanced resource recovery efficiency achieved by the coagulant dosing (Exp. 4) seems not to overcome the expenses of the chemicals involved, requiring slight economic inputs despite the higher energy recovery (see Table 6). However, since no great differences were obtained between the economic impact of Exp. 2 and Exp. 4, the use of coagulants can still be recommended as an interesting strategy to boost the DM formation capacity and increase resource recovery. Moreover, other side effects such as higher phosphate recovery or the environmental impact of using these chemicals should also be considered. Thus, further studies are needed to properly assess the suitability of dosing coagulant in this alternative treatment scheme. Regarding the operating TSS concentration, this study showed that although increasing them can favour DM self-forming time, important enhancements of resource capture efficiency cannot be obtained in the short-term. This strategy would thus negatively affect the required filtration energy due to the significant increase in operating TMP. Since coagulant dosing can significatively reduce DM forming time while improving resource capture capacity, relatively low operating TSS could be recommended when operating a DM for treating MWW.

Comparing the results obtained in this work with other studies using DMs to treat raw MWW, significantly lower energy recoveries were achieved (see Table 6), only reaching similar results when coagulant was dosed. These results were attributed to the different influent used in this study, since raw MWW have a higher number of recoverable resources while in the proposed alternative, the primary settler recovers a significant fraction of these resources. Thus, taking into account that about 50% of the raw influent TSS would be recovered in the primary settler, the overall energy outputs achieved by the proposed alternative would increase to 0.215–0.308 kWh per m^3^ of treated MWW, values higher than the energy recovery reported when directly filtering raw MWW. Since a lower fouling rate could be expected when filtering more treated influents due to the reduced fraction of influent pollutants, the proposed alternative could be an interesting approach to boost resource recovery while reducing the required filtration energy.

In addition to the discussion made in this section, other considerations need to be taken into account to properly choose the most suitable DM operating conditions. Fouling development during DM operation should be carefully controlled by employing continuous physical cleaning methodologies (e.g., air scouring), determining the optimum conditions to minimize filtration energy requirements without compromising DM integrity or permeate quality. The operating TSS should also be optimized not only considering the energy required for filtration, but also the subsequent use of the concentrated sludge (i.e., methane production via anaerobic digestion). Thus, all the extra steps and full energy requirements for using this sludge should also be considered (pumping demands, sludge thickening, etc.) to determine the most feasible operating conditions for the overall process. Similarly, permeate quality should be adjusted according to its foreseen use (direct discharge to water bodies, tertiary wastewater treatments, etc.), which could significantly influence the proper operating flux or coagulant dosing. On the other hand, other improvements could be made concerning the membrane operating parameters. In this study, a high waste/influent operating ratio was used in order to avoid a high sludge retention time in the membrane tank, which would be an undesirable full-scale operating condition due to the high flow rate of the produced waste. Thus, reducing the membrane tank volume as much as possible would be an important design strategy for boosting the energy and economic balances of this technology, as it would reduce the waste stream to treat while increasing its TSS and COD concentration, significantly reduce the membrane tank sludge retention time and also reduce the process space requirements.

It can thus be concluded that treating PSE by DM can be considered an interesting alternative within the DMF approach to improve resource recovery from MWW while reducing process energy requirements. However, further studies, considering all the above exposed and comparing the results obtained with other membrane technologies (e.g., other supporting materials, MF and UF membranes, etc.) and influents (e.g., raw MWWs) need to be performed to properly determine the best scenarios for full-scale implementation of the proposed alternative.

## 4. Conclusions

DM feasibility for treating MWW was evaluated in this study. The main findings were as follows:

- One layer of the supporting material (a flat open monofilament woven polyamide mesh of 1 µm average pore size) was not enough to self-form a DM in the short-term when treating PSE from a full-scale WWTP, showing the limitations of DMs for treating more depurated influents. Nevertheless, a proper DM was self-formed when using two supporting material layers (17 days of operation) or when increasing the operating TSS concentration (8, 6 and 4 days of operation for a TSS concentration of 1.9, 4.7 and 9.2 g L^−1^).
- Similar permeate qualities were obtained regardless of filtration flux and TSS tested in this study, achieving TSS, COD, TN, TP and turbidity values of 65 mg L^−1^, 141 mg L^−1^, 42.3 mg L^−1^, 4.3 mg L^−1^ and 86 NTU, respectively.
- Coagulant dosing improved both the required forming time and DM permeate quality. Optimum coagulant (PHLA18) dosing of 10 mg L^−1^ was determined, achieving a DM forming time of 7 days and a permeate quality of TSS, COD, TN, TP and turbidity of 24 mg L^−1^, 58 mg L^−1^, 38.1 mg L^−1^, 1.2 mg L^−1^ and 22 NTU, respectively.
- Preliminary energy and economic balances showed that energy recoveries from 0.032 to 0.121 kWh per m^3^ of treated water at an economic cost of from €0.002 to €0.003 per m^3^ of treated water can be obtained from the recovered particulate material.

## Figures and Tables

**Table 1:** Influent characteristics.

Parameter	Units	Mean ± SD
TSS	mg TSS L^−1^	113 ± 22
COD	mg COD L^−1^	167 ± 42
SCOD	mg COD L^−1^	57 ± 21
TN	mg N L^−1^	45.5 ± 8.5
TP	mg P L^−1^	5.9 ± 1.1
Alk	mg CaCO_3_ L^−1^	335 ± 67
pH	-	7.6 ± 0.5
Turbidity	NTU	109 ± 31

**Table 2:** Experimental setup.

**PILOT-PLANT**			
**Exp.**	**Supporting Material**	Operating Flux (LMH) *	**Coagulant Concentration** **(mg Al_2_O_3_ L^−1^)**
1	1 layer	15.4 ± 0.2	-
2	2 layers	15.1 ± 0.3	-
3	2 layers	45.3 ± 3.1	-
4	2 layers	14.7 ± 0.7	10
**LAB-SCALE**			
**Exp.**	**Supporting Material**	Operating Flux (LMH) *	**Sludge Concentration** **(g L^−1^)**
1L	1 layer	15.3 ± 0.6	1.9
2L	1 layer	14.8 ± 0.6	4.7
3L	1 layer	14.9 ± 0.9	9.2

* Operating flux corrected to a temperature of 20 °C.

**Table 3:** Features of the different coagulants used in this study (Feralco Iberia S.A.).

Coagulant	Product Code	Formula	% Al_2_O_3_	% Cl^−^	% SO_4_^2−^
1	PHAL 18	Al(OH)_a_Cl_b_	17.0 ± 0.5	21.5 ± 1.0	-
2	PHAL 10	Al(OH)_a_Cl_b_(SO_4_)_c_	10.0 ± 0.3	12.0 ± 0.5	2.1 ± 0.2
3	AQUALENC F1	Al(OH)_a_Cl_b_(SO_4_)_c_	9.5 ± 0.5	12.3 ± 1.3	n.a.
4	AQUALENC F2	Al(OH)_a_Cl_b_(SO_4_)_c_	9.0 ± 0.5	10.5 ± 0.5	1.5 ± 0.5

All compounds are expressed in mass percentages. n.a.: not available.

**Table 4:** Permeate quality.

Exp.	TSS		Turbidity		COD		TN		TP	
	(mg L^−1^)	(%) *	(NTU)	(%) *	(mg L^−1^)	(%) *	(mg L^−1^)	(%) *	(mg L^−1^)	(%) *
2	65	58	86	79	141	84	42.3	93	4.3	73
3	59	52	94	86	138	83	42.9	94	4.4	75
4	24	21	22	20	58	35	38.1	84	1.2	20

* Percentage of the influent pollutant remaining in the permeate.

**Table 5:** Average transmembrane pressure (TMP) after the dynamic membrane formation.

Exp.	Sludge Concentration (g L^−1^)	Operating Days	Self-Forming Period (Days)	Average TMP (mbar)
1L	1.9	15	8	56
2L	4.7	15	5	198
3L	9.2	6	4	384

**Table 6:** Energy and operating cost of DM operation.

Exp. Num	MWW Treated	Energy Recovery (kWh m^−3^)	Energy Costs (€ m^−3^)	Coagul. Costs (€ m^−3^)	Costs Output (€ m^−3^)	Reference
2	PSE	0.029	−0.002	-	−0.002	This study
3	PSE	0.032	−0.002	-	−0.002	This study
4	PSE	0.121	−0.009	0.012	0.003	This study
-	Raw	0.101	n.a.	n.a.	n.a.	[10]
-	Raw	0.127	n.a.	n.a.	n.a.	[12]

n.a.: not available.

## Data Availability

Data is contained within the article.

## References

[1] Batstone D., Hülsen T., Mehta C., Keller J. (2015). Platforms for energy and nutrient recovery from domestic wastewater: A review. Chemosphere.

[2] Puyol D., Batstone D.J., Hülsen T., Astals S., Peces M., Krömer J. (2017). Resource Recovery from Wastewater by Biological Technologies: Opportunities, Challenges, and Prospects. Front. Microbiol..

[3] Sid S., Volant A., Lesage G., Heran M. (2017). Cost minimization in a full-scale conventional wastewater treatment plant: Associated costs of biological energy consumption versus sludge production. Water Sci. Technol..

[4] Vinardell S., Astals S., Peces M., Cardete M.A., Fernández I., Mata-Alvarez J., Dosta J. (2020). Advances in anaerobic membrane bioreactor technology for municipal wastewater treatment: A 2020 updated review. Renew. Sustain. Energy Rev..

[5] Zhao Y.-X., Li P., Li R.-H., Li X.-Y. (2019). Direct filtration for the treatment of the coagulated domestic sewage using flat-sheet ceramic membranes. Chemosphere.

[6] Hube S., Eskafi M., Hrafnkelsdóttir K.F., Bjarnadóttir B., Bjarnadóttir M.Á., Axelsdóttir S., Wu B. (2020). Direct membrane filtration for wastewater treatment and resource recovery: A review. Sci. Total Environ..

[7] Kimura K., Honoki D., Sato T. (2017). Effective physical cleaning and adequate membrane flux for direct membrane filtration (DMF) of municipal wastewater: Up-concentration of organic matter for efficient energy recovery. Sep. Purif. Technol..

[8] Jin Z., Gong H., Temmink H., Nie H., Wu J., Zuo J., Wang K. (2016). Efficient sewage pre-concentration with combined coagulation microfiltration for organic matter recovery. Chem. Eng. J..

[9] Nascimento T.A., Fdz-Polanco F., Peña M. (2020). Membrane-Based Technologies for the Up-Concentration of Municipal Wastewater: A Review of Pretreatment Intensification. Sep. Purif. Rev..

[10] Xiong J., Yu S., Hu Y., Yang Y., Wang X. (2019). Applying a dynamic membrane filtration (DMF) process for domestic wastewater preconcentration: Organics recovery and bioenergy production potential analysis. Sci. Total Environ..

[11] Gong H., Wang X., Zheng M., Jin Z., Wang K. (2014). Direct sewage filtration for concentration of organic matters by dynamic membrane. Water Sci. Technol..

[12] Ma J., Wang Z., Xu Y., Wang Q., Wu Z., Grasmick A. (2013). Organic matter recovery from municipal wastewater by using dynamic membrane separation process. Chem. Eng. J..

[13] Usman M., Belkasmi A.I., Katsoyiannis I.A., Ernst M. (2021). Pre-deposited dynamic membrane adsorber formed of microscale conventional iron oxide-based adsorbents to remove arsenic from water: Application study and mathematical modeling. J. Chem. Technol. Biotechnol..

[14] Hu Y., Wang X.C., Ngo H.H., Sun Q., Yang Y. (2018). Anaerobic dynamic membrane bioreactor (AnDMBR) for wastewater treatment: A review. Bioresour. Technol..

[15] Mohan S.M., Nagalakshmi S. (2020). A review on aerobic self-forming dynamic membrane bioreactor: Formation, performance, fouling and cleaning. J. Water Process Eng..

[16] Hey T., Bajraktari N., Davidsson Å., Vogel J., Madsen H.T., Hélix-Nielsen C., Jansen J.L.C., Jönsson K. (2018). Evaluation of direct membrane filtration and direct forward osmosis as concepts for compact and energy-positive municipal wastewater treatment. Environ. Technol..

[17] Gong H., Jin Z., Wang X., Wang K. (2015). Membrane fouling controlled by coagulation/adsorption during direct sewage membrane filtration (DSMF) for organic matter concentration. J. Environ. Sci..

[18] Te Poele S. (2006). Foulants in Ultrafiltration of Wwtp Effluent. Ph.D. Thesis.

[19] Ravazzini A.M. (2008). Crossflow Ultrafiltration of Raw Municipal Wastewater. Ph.D. Thesis.

[20] Ravazzini A., van Nieuwenhuijzen A., van der Graaf J. (2005). Direct ultrafiltration of municipal wastewater: Comparison between filtration of raw sewage and primary clarifier effluent. Desalination.

[21] APHA, AWWA, WEF (2012). Standard Methods for the Examination of Water andWastewater.

[22] Darrow K., Tidball R., Wang J., Hampson A. Catalog of CHP Technologies, U.S. Environmental Protection Agency Combined Heat and Power Partnership; at ICF International (September 2017), with Funding from the U.S. Environmental Protection Agency and the U.S. Department of Energy. https://www.epa.gov/sites/default/files/2015-07/documents/catalog_of_chp_technologies.pdf.

[23] (2021). Gesternova Energía, Spanish Electricity Rates (Tarifa Eléctrica España). https://gesternova.com/tarifas-luz/tarifas-luz-alta-tension/.

[24] (2021). Aura Energía, Spanish Electricity Rates (Tarifa Eléctrica España). https://www.aura-energia.com/tarifas-luz-industria-peninsula/.

[25] Jiang L., Yu Y., Liu G. (2021). Effects of inorganic particles and their interactions with biofilms on dynamic membrane structure and long-term filtration performance. Sci. Total Environ..

[26] Yu W., Xu L., Qu J., Graham N. (2014). Investigation of precoagulation and powder activate carbon adsorption on ultrafil-tration membrane fouling. J. Membr. Sci..

[27] Hey T. (2016). Municipal Wastewater Treatment by Microsieving, Microfiltration and Forward Osmosis. Concepts and Potentials. Ph.D. Thesis.

[28] Chen Y., Wu Y., Wang D., Li H., Wang Q., Liu Y., Peng L., Yang Q., Li X., Zeng G. (2018). Understanding the mechanisms of how poly aluminium chloride inhibits short-chain fatty acids production from anaerobic fermentation of waste activated sludge. Chem. Eng. J..

[29] Hafuka A., Takahashi T., Kimura K. (2020). Anaerobic digestibility of up-concentrated organic matter obtained from direct membrane filtration of municipal wastewater. Biochem. Eng. J..

